# Causal relationship between lymphocyte subsets and the risk of sepsis: A Mendelian randomization study

**DOI:** 10.1097/MD.0000000000039871

**Published:** 2024-10-04

**Authors:** Jing Chen, Rong Hui Wang, Sheng Xie, Jun Jun Xiang, Fu Kui Zheng, Qiao Ming Huang, Qiu Lan Mo, Qiu Gui Wei, Zu Lu Liu

**Affiliations:** a The First Affiliated Hospital of Guangxi University of Traditional Chinese Medicine, Nanning, China.

**Keywords:** causal relationship, lymphocyte subsets, randomization, sepsis, T cell.

## Abstract

Recent empirical research posits a link between lymphocyte subgroups and both the incidence and prognosis of sepsis. Nevertheless, the potential influence of multiple confounding variables obscures any clear causative correlation. Utilizing a 2-sample Mendelian randomization approach, we conducted a meta-analysis of lymphocyte subgroups. In a genome-wide association study, flow cytometry was applied to a lymphocyte subgroup comprising 3757 Sardinians to identify genes influenced by blood immune cells. The sepsis meta-analysis data were sourced from the UK Biobank database, including 11,643 treatment groups and 47,841 control groups. Inverse variance-weighted, Mendelian randomization-Egger regression, weighted median, simple mode, and weighted mode methods were deployed to ascertain the causative relationship between lymphocyte subgroup and sepsis. Cochran *Q* test, the Mendelian randomization-Egger intercept test, and funnel plots were leveraged to assess the robustness of study findings. The inverse variance-weighted analysis disclosed that the absolute count of CD4 regulatory T cells (CD4 Treg AC) within the lymphocyte subgroup has a causative link to an elevated risk of sepsis, with an odds ratio of 1.08 and a 95% confidence interval of 1.02 to 1.15 (*P* = .011). Compared to individuals not subjected to this factor, those exposed to CD4 Treg AC have a marginally elevated sepsis risk by approximately 0.08%. No causative relationships were observed between sepsis risk and the absolute counts of other lymphocyte subgroups such as CD8+ T cells, CD4+ CD8dim T cells, natural killer T cells, B cells (B cell absolute count), and HLA DR+ natural killer cells. The 2-sample Mendelian randomization study indicated a causal relationship between the level of CD4 Treg AC and the increased risk of sepsis. The elevation in circulating lymphocyte subgroups suggests higher susceptibility to sepsis, affirming the immune susceptibility inherent to this condition. The findings from our study may propose potential targets for diagnosis and intervention of sepsis.

## 1. Introduction

Sepsis, a life-threatening organ dysfunction resulting from a dysregulated host response to infection, carries high morbidity, mortality, and poor prognosis.^[1,2]^ Annually, there are over 30 million sepsis patients worldwide, leading to more than 5 million deaths. The incidence of sepsis has a deep correlation with the economic state of a country- low-income countries report a significantly higher incidence than their high-income counterparts.^[3]^ Sepsis remains a major global health concern, placing a substantial economic burden on families and societies. Existing treatment strategies for sepsis include anti-infection measures, bedside blood purification, fluid resuscitation, organ protection, and immune boosting, among others.^[4]^ The pathogenesis of sepsis is associated with multiple factors, including immune dysfunction, endotoxin translocation, and secretion of inflammatory factors.^[5]^ Survivors of the initial inflammatory stage of sepsis often exhibit postsepsis immune suppression, making them susceptible to opportunistic secondary infections.^[6]^ Abnormal immune regulation plays a critical role in the onset and progression of sepsis.^[7]^ It’s observed that both innate and adaptive immunity components often malfunction in sepsis patients.^[8]^

Lymphocytes, long regarded as a significant element of the dysregulated immune response in critical illnesses, particularly sepsis, can be functionally diverse despite their morphological uniformity.^[9]^ They can be divided into 3 types based on their biological functions and variations in cell surface antigen expression: T cells, B cells, and natural killer cells (NK cells).^[10]^ Integral to the acquired immune response to infection, lymphocytes and their subgroups greatly influence sepsis outcomes and patient survival.^[11]^ Sepsis-related immune suppression commonly manifests as lymphocyte function impairment and an increased expression of inhibitory checkpoint molecules, such as programmed death protein 1.^[6]^ Research confirms that the up-regulation of PD-PD-L1 levels in T cells, monocytes, and neutrophils elevates mortality rates.^[12–14]^ However, the correlation between lymphocyte subgroups and sepsis observed in studies can be convoluted by multiple confounding factors, posing a comprehensive control challenge within these studies.

Mendelian randomization (MR) is a statistical method grounded on whole-genome sequencing data, employed to discern causal relationships. Katan proposed in 1986^[15]^ that distinct genotypes engender discrete intermediate phenotypes. In circumstances where the phenotype embodies certain individual exposure characteristics, the associative effect between genotype and disease can exemplify the impact of the exposure factor on the disease. While bearing similarities to randomized controlled trial research, MR notably circumvents the limitations of observational studies, which are prone to influence by confounding or reverse causality, thereby curbing bias. This methodological advantage has facilitated extensive uses of MR to probe the causal links between circulating immune cells and various diseases, including metabolic diseases.^[16]^

## 2. Materials and methods

### 2.1. Study design

**Figure 1.**
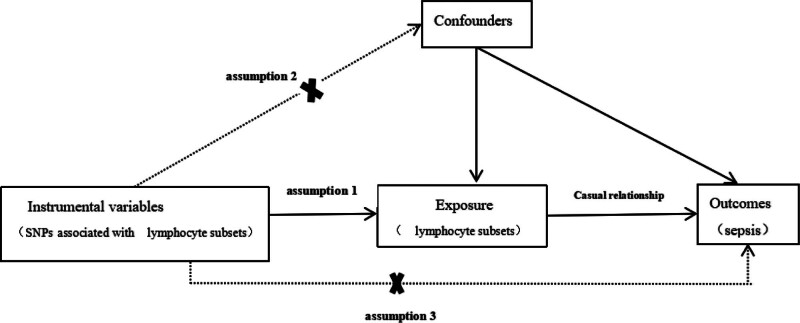
Overview of the overall MR design. Lymphocyte subsets include CD4 Treg AC, CD8+ T cell AC, CD4+CD8dim T cell AC, NK TAC, HLA DR+ NK AC, and B cell AC. B cell AC = B cell absolute count, CD4 Treg AC = CD4 regulatory T cell absolute count, CD8+ T cell AC = CD8+ T cell Absolute Count, CD4+CD8dim T cell AC = CD4+ CD8dim T cell absolute count, NK TAC = Natural Killer T Absolute Count, HLA DR+ NK AC = HLA DR+ natural killer absolute count, MR = Mendelian randomization.

**Figure 2.**
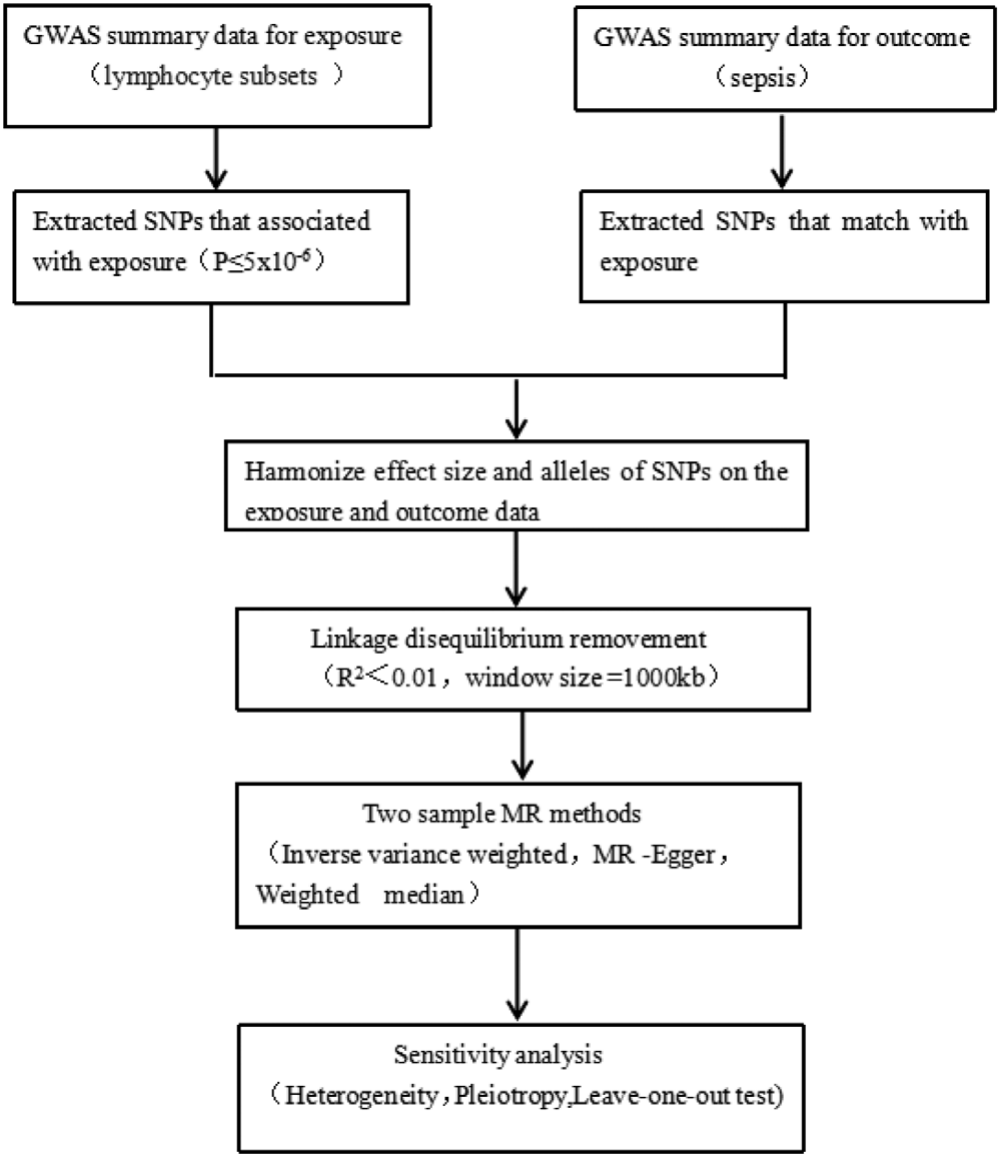
Study design and workflow. GWAS = genome-wide association study, MR = Mendelian randomization, SNP = single nucleotide polymorphisms.

In this research, we selected serum lymphocyte subgroups (CD4 regulatory T cell absolute count [CD4 Treg AC], CD8+ T cell AC (Absolute Count), CD4+ CD8dim T cell absolute count [CD4+CD8dim T cell AC], natural killer T absolute count [NKT AC], HLA DR+ natural killer absolute count [HLA DR+ NK AC], B cell absolute count [B cell AC]) as exposure factors. We used single nucleotide polymorphisms (SNPs) significantly associated with these exposure factors to serve as instrumental variables (IVs), and took sepsis as the outcome variable. An MR analysis was conducted using summary-level public data from a genome-wide association study (GWAS) to evaluate the causality between the lymphocyte subgroups and sepsis risk. Cochran *Q* test was employed to assess heterogeneity and a series of sensitivity analyses were undertaken to affirm the trustworthiness of the causal association findings. To conduct an MR analysis, 3 core assumptions must be fulfilled^[17]^: the IV has a tight connection with exposure, it is independent of exposure and outcome’s confounding factors,^[18]^ it only affects the outcome through the exposure. The specified 2-sample MR study model and flowchart utilized in this research can be found in Figures 1 and 2. See Figure 1, Supplemental Digital Content, http://links.lww.com/MD/N657, which shows specific information on 6 exposure factors, see details of Figure 1-a, Figure 1-b, Figure 1-c, Figure 1-d, Figure 1-e, and Figure 1-f.

### 2.2. Data sources

The data summarized for this 2-sample MR study is derived from the Iatrogenic Europe Unite Open GWAS database (https://gwas.mrcieu.ac.uk/). This study presents genetic association estimates between lymphocyte subgroups and SNPs extracted from a GWAS,^[19]^ which conducted flow cytometry analysis on lymphocyte subgroups consisting of 3757 Sardinians. The exposure factors include CD4 Treg AC (GWAS ID:ebi-a-GCST90001513); CD8+ Tcell AC (GWAS ID:ebi-a-GCST90001592); CD4+CD8dim T cell AC (GWAS ID:ebi-a-GCST90001609); NKT AC (GWAS ID:ebi-a-GCST90001621); B cell AC (GWAS ID:ebi-a-GCST90001642); and HLA DR+ NK AC (GWAS ID:ebi-a-GCST90001648). The outcome of interest is sepsis (GWAS ID: ieu-b-4980), represented by data from the UK Biobank database, encompassing 486,484 individuals with sepsis, split into 11,643 cases and 47,841 controls. The aforementioned databases source their information from European populations. All datasets utilized in this study are publicly available. See Table 1, which shows summary of the GWAS included in this 2-sample MR study.

**Table 1:** Summary of the GWAS included in this 2-sample MR study.

Variable	ID	Sample size	Number of SNPS	Consortium	Population	Sex	Year
CD4 regulatory T cell absolute count	ebi-a-GCST90001513	3405	15,131,843	NA	European	Males and females	2020
CD8+ T cell absolute count	ebi-a-GCST90001592	3652	15,195,743	NA	European	Males and females	2020
CD4+ CD8dim T cell absolute count	ebi-a-GCST90001609	3652	15,195,743	NA	European	Males and females	2020
HLA DR+ natural killer absolute count	ebi-a-GCST90001648	3580	15,158,016	NA	European	Males and females	2020
Natural killer T absolute count	ebi-a-GCST90001621	3653	15,195,758	NA	European	Males and females	2020
B cell absolute count	ebi-a-GCST90001642	3653	15,195,758	NA	European	Males and females	2020
Sepsis	ieu-b-4980	486,484	12,243,539	UK Biobank	European	Males and females	2021

GWAS = genome-wide association study, MR = Mendelian randomization, NA = not available, SNPs = single-nucleotide.

### 2.3. Selection of IVs

This study followed a 3-step process to meet the MR assumptions. First, to ensure SNPs were strongly linked to the exposure factor, we identified SNPs with a significant whole-genome-level association with the exposure factor. Guided by the principle of preventing inaccurate results due to limited SNPs, we selected genetic IVs^[20]^ based on a somewhat relaxed threshold (*P* < 5 × 10^−6^). Second, to confirm the independent IVs exposure, we applied an *R*^2^ cutoff of 0.01 and a kilobase pairs (kb) limit of 1000 to eradicate the potential interference of linkage disequilibrium.^[21]^ Finally, we used the effect allele frequency to harmonize the respective exposure and outcome datasets, concurrently eliminating palindromic SNPs having intermediate allele frequencies. To assess the potential weak IV bias of our selected IVs, we computed the *F*-statistic; an *F*-statistic >10 indicates the absence of a weak IV bias. The following formula represents the computation of the *F*-statistic for each SNP: 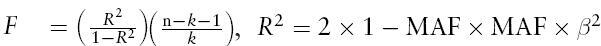.The variable “n” denotes the sample size of the lymphocyte subgroup, while “*k*” symbolizes the quantity of SNPs. “*R*^2^” signifies the percentage of variations attributed to SNPs within the lymphocyte subgroup database. “*R*^2^” is calculated using the formula *R*^2^ = 2 × EAR × (1 − EAF) × *β*^2^, in which EAF represents the effect allele frequency, and *β* symbolizes the allele effect value.^[22]^ In this study, the *F*-value serves as a statistical metric, employed to assess the impact of weak IVs. An *F*-value exceeding 10 correlates to each individual SNP we studied, insinuating the absence of weak IVs bias in the outcomes. The PhenoScanner V2 website database (http://www.phenoscanner.medschl.cam.ac.uk/) was used to inquire and omit SNPs correlated with confounding factors.

### 2.4. Statistical analysis for MR

Five methods were employed in this study to estimate the causal relationship between lymphocyte subgroups and sepsis. These included the inverse variance-weighted (IVW) method,^[23]^ MR-Egger regression,^[24]^ weighted median estimator,^[25]^ simple mode,^[26]^ and weighted mode method.^[27]^ IVW, serving as the primary analysis method for evaluating causal relationships in MR research, is the most commonly used. The remaining 4 methods supplement IVW. MR-Egger leverages the intercept to gauge pleiotropy. An intercept value equal to zero indicates no horizontal pleiotropy, and consistency with IVW in the MR-Egger regression result.^[24]^ In scenarios with up to 50% invalid IVs and significant variance in estimate precision, the weighted median estimator,^[25]^ designated as the median of the ratio estimate of the weighted empirical density function, can provide consistent effect estimates. The MR analyses were executed using the “TwoSampleMR” package (version 0.5.6) within the R software environment (version 4.1.2).^[28]^

### 2.5. Sensitivity analysis

To ensure the reliability of our study results, we carried out a number of sensitivity analyses, specifically Cochran *Q* test, MR-Egger intercept test, and a funnel plot. Cochran *Q* test indicated the presence of genetic heterogeneity among the IVs utilized for measuring lymphocyte subgroups. We examined the heterogeneity of the primary IVW and MR-Egger methods via the Cochran *Q* statistic and *P* values, with a derived *P* < .05 from Cochran *Q* marking the presence of heterogeneity. Horizontal pleiotropy was assessed using the intercept of the MR-Egger regression (*P* < .05 implies the presence of horizontal pleiotropy).^[29]^ We employed the “leave-one-out” method to omit each SNP and subsequently conducted an MR analysis to identify outliers, primarily examining whether the means are exclusively on 1 side of 0 or 1. In the end, funnel plots were used to manually check the symmetric distribution of SNPs on either side of the IVW line. The scatter plot of the causal association effect appeared largely symmetrical, suggesting scarce potential bias in the results. To further mitigate horizontal pleiotropy, I applied MR-PRESSO analysis, primarily aimed at reducing horizontal pleiotropy by eliminating significant outliers.^[30]^

## 3. Results

### 3.1. The result of IVs

In examining the correlation between levels of lymphocyte subgroups and sepsis, we selected and coordinated the IVs. Assumption 1, instrument variables are robustly related to exposure; assumption 2, instrument variables are not related to confounders; assumption 3, instrument variables are related to outcome only through exposure. The specified 2-sample MR study model in this research can be found in Figure 1.The statistical results of *F* show that all *F* values are >10, which conforms to the strong correlation hypothesis.

We acquired SNPs respective to lymphocyte subgroups at the levels of CD4 Treg AC, CD8+ T cell AC, CD4+CD8dim T cell AC, NKT AC, B cell AC, HLA DR+ NK AC. The amounts of SNPs observed were 11, 12, 9, 24, 11, and 9 with *P* < 5 × 10^−6^. The *F*-statistics for each SNP ranged from 21.38 to 136.64, indicating bias from weak IVs was removed.

### 3.2. MR analysis results

MR analysis results interpret the odds ratio (OR) as follows: an OR >1 denotes that the exposure is deemed a risk factor for the outcome, while an OR <1 labels the exposure as a protective factor against the outcome. Statistical significance is affirmed when *P* is <.05. Table 2 reveals that CD4 Treg antigen complex (AC) is a statistically significant risk factor for sepsis, with the IVW result for CD4 Treg AC yielding an OR of 1.08 (95% confidence interval = 1.02–1.15, *P* = .011). This suggests that subjects exposed to CD4 Treg AC are approximately 0.08% more likely to develop sepsis compared to those not exposed. However, the risk of sepsis showed no significant association with the absolute numbers of other cell types, such as CD8+ T cell absolute count, CD4+CD8dim T cell AC, NKT AC, B cell AC, and HLA DR+ NK AC.

**Table 2:** The Mendelian randomization analysis results with regard to causal effect of lymphocyte subsets on sepsis.

Exposure	SNPs (n)	IVW			WME		MR-Egger	
		OR (95% CI)	*Q* (*P* value)	*P* value	OR (95% CI)	*P* value	OR (95% CI)	*P* value
CD4 regulatory T cell absolute count	11	1.08 (1.02–1.15)	6.63 (0.760)	0.011	1.08 (0.97–1.21)	0.206	1.08 (0.94–1.24)	0.324
CD8+ T cell absolute count	12	1.00 (0.97–1.04)	13.1 (0.285)	0.804	1.03 (0.97–1.08)	0.359	1.01 (0.9–1.07)	0.628
CD4+ CD8dim T cell absolute count	9	1.02 (0.96–1.09)	8.68 (0.370)	0.466	1.01 (0.94–1.09)	0.771	1.01 (0.8–1.18)	0.924
HLA DR+ natural killer absolute count	9	0.96 (0.91–1.01)	9.76 (0.282)	0.12	0.92 (0.86–0.98)	0.029	0.89 (0.8–0.96)	0.017
Natural killer T absolute count	24	0.99 (0.96–1.03)	23.3 (0.444)	0.680	1.02 (0.95–1.09)	0.650	1.00 (0.94–1.07)	0.924
B cell absolute count	11	0.95(0.88–1.02)	12.3 (0.265)	0.129	0.93 (0.84–1.03)	0.219	0.94 (0.79–1.13)	0.528

CI = confidence interval, IVW = inverse variance weighted, OR = odds ratio, SNP = single nucleotide polymorphisms, WME = weighted mode methods.

### 3.3. Sensitivity analysis results

**Figure 3.**
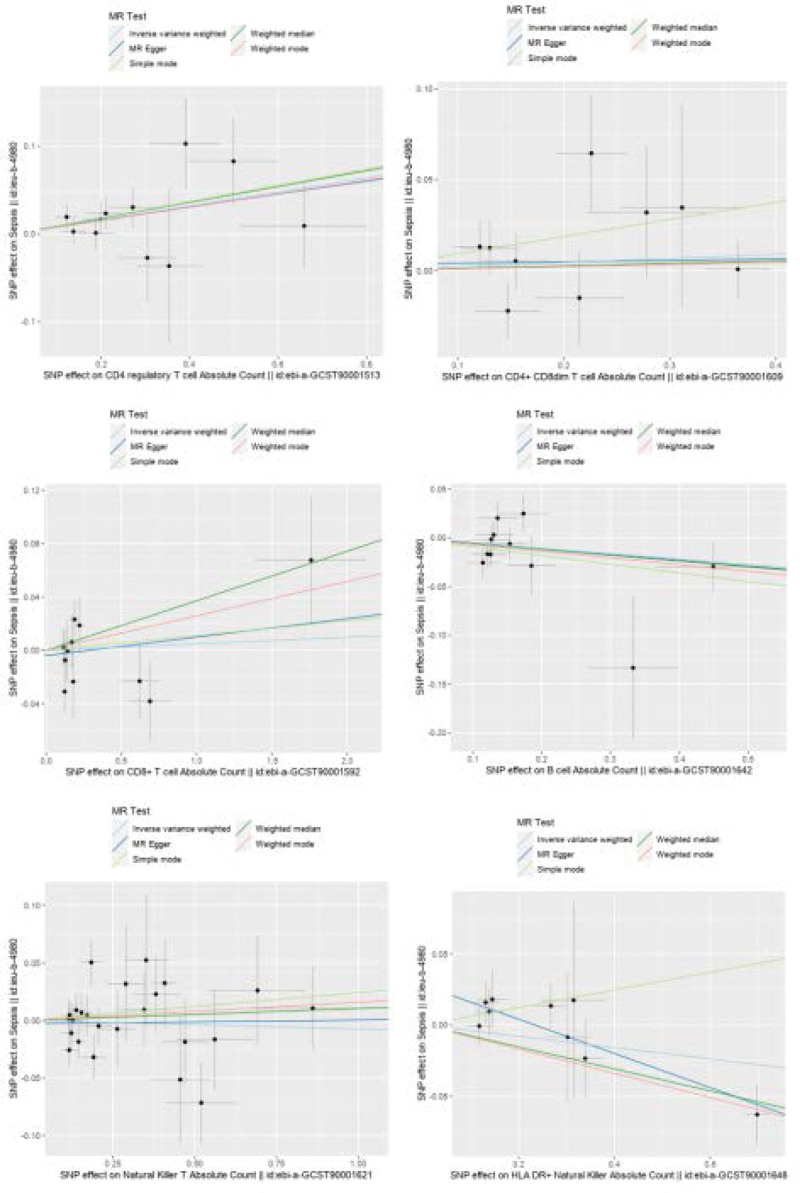
Panel (A) represents the CD4 regulatory T cell absolute count in relation to sepsis; panel (B) represents the CD4+ CD8dim T cell absolute count in relation to sepsis; panel (C) represents the CD8+ T cell absolute count in relation to sepsis; panel (D) represents the B cell absolute count in relation to sepsis; panel (E) represents the natural killer T absolute count in relation to sepsis; panel (F) represents the HLA DR+ natural killer absolute count in relation to sepsis. Black dots indicate SNPs; vertical and horizontal line segments indicate the range of SNP effects; 5 diagonal line segments represent the 5 tools of Mendelian randomization analysis. If the diagonal line is upward, it indicates a positive causal relationship between the exposure factor and the outcome, whereas if it is downward, the opposite is true. SNP = single nucleotide polymorphisms.

**Figure 4.**
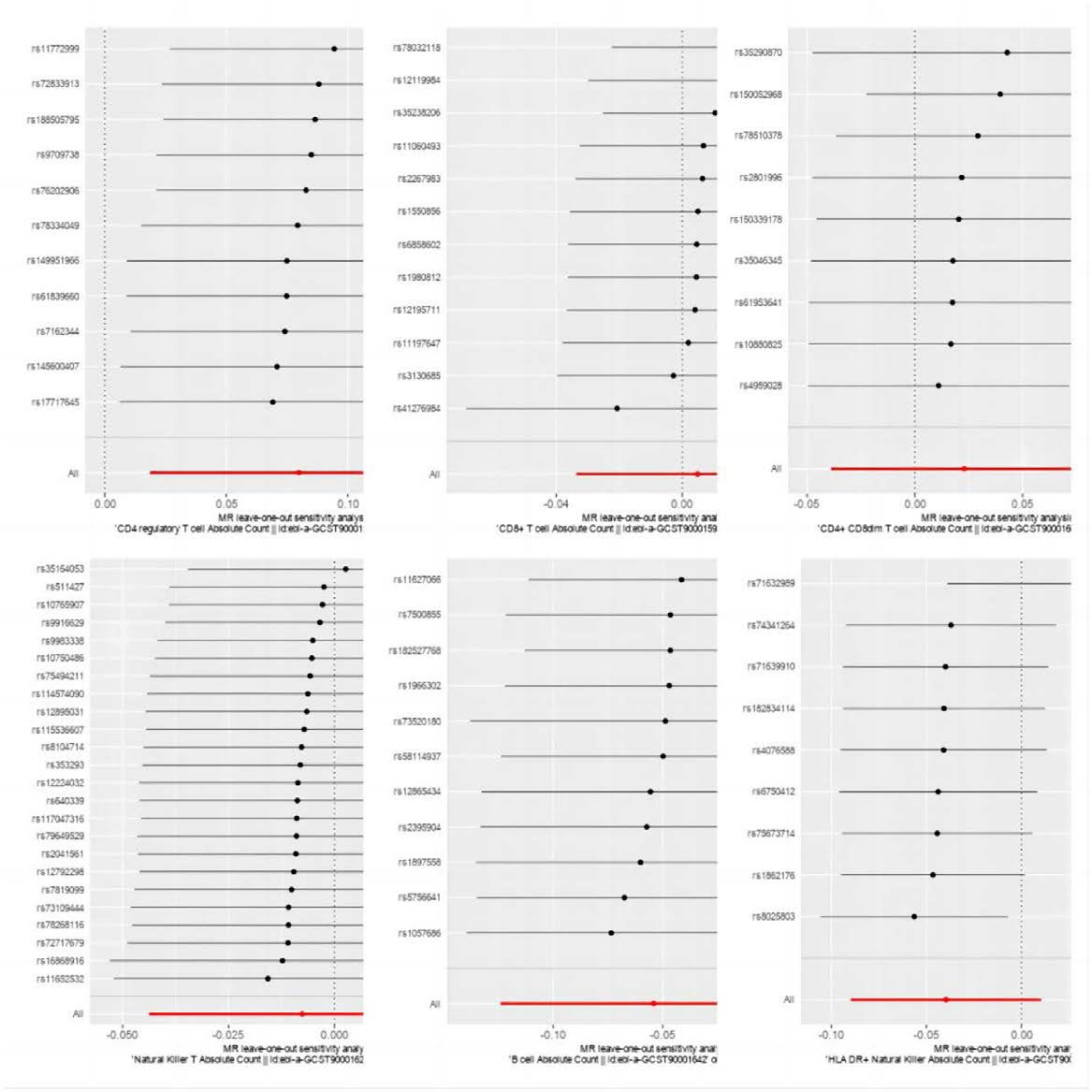
The horizontal axis represents the regression coefficient, and the vertical axis represents the SNP identifier. Panel (A) depicts the Mendelian randomization sensitivity analysis of “CD4 regulatory T cell Absolute Count” with respect to “sepsis”; panel (B) depicts the Mendelian randomization sensitivity analysis of “CD8+ T cell Absolute Count” with respect to “sepsis”; panel (C) depicts the Mendelian randomization sensitivity analysis of “CD4+ CD8dim T cell Absolute Count” with respect to “sepsis”; panel (D) depicts the Mendelian randomization sensitivity analysis of “Natural Killer T Absolute Count” with respect to “sepsis”; panel (E) depicts the Mendelian randomization sensitivity analysis of “B cell Absolute Count” with respect to “sepsis”; panel (F) depicts the Mendelian randomization sensitivity analysis of “HLA DR+ Natural Killer Absolute Count” with respect to “sepsis.” SNP = single nucleotide polymorphisms.

**Figure 5.**
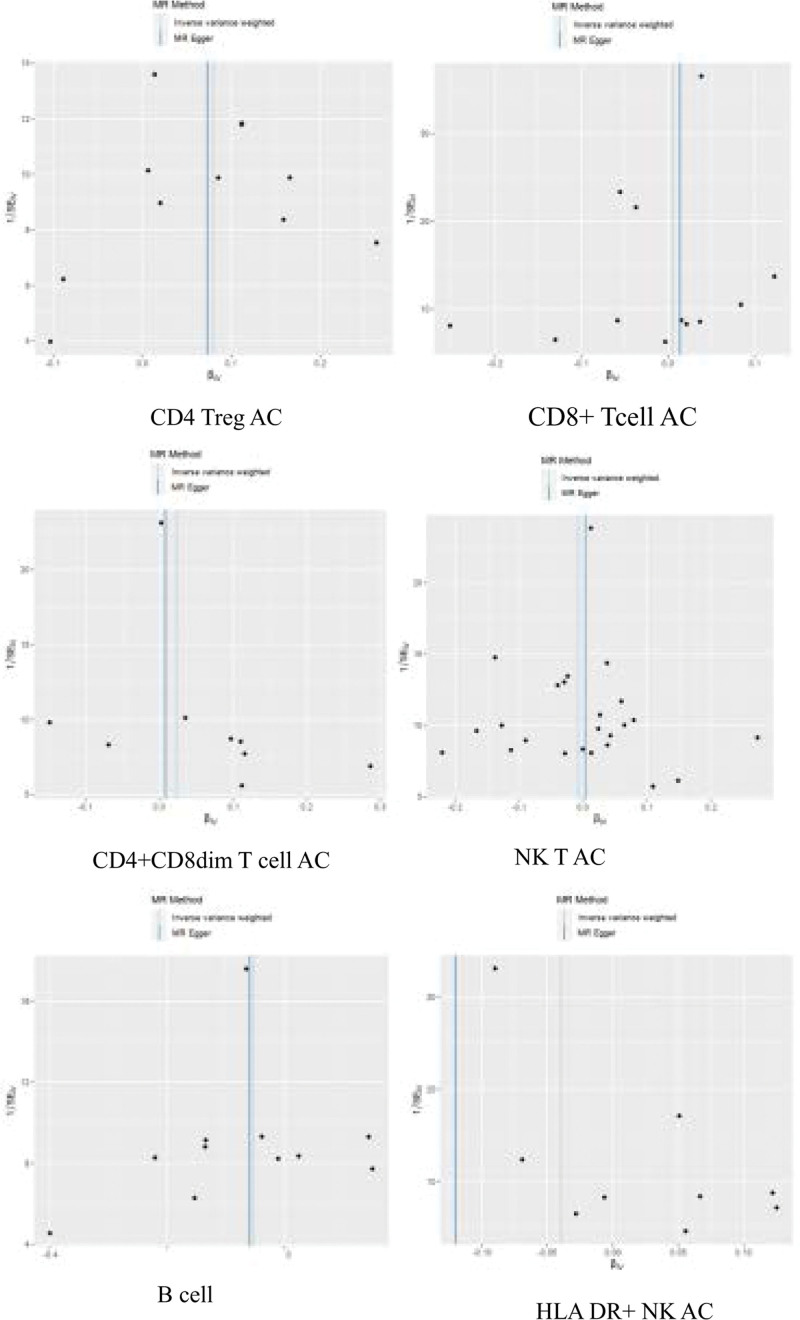
Panel (A) represents the CD4 regulatory T cell absolute count in relation to sepsis; panel (B) represents the CD8+ T cell absolute count in relation to sepsis; panel (C) represents the CD4+ CD8dim T cell absolute count in relation to sepsis; panel (D) represents the natural killer T absolute count in relation to sepsis; panel (E) represents the B cell absolute count in relation to sepsis; panel (F) represents the HLA DR+ natural killer absolute count in relation to sepsis. The X-axis represents the beta value of the instrumental variable SNP, and the Y-axis represents the standard error of the instrumental variable SNP. Black dots represent SNPs. When 2 vertical lines are close to the centerline and symmetrical dots are observed on both sides, it is considered that there is no heterogeneity in the Mendelian randomization analysis. SNP = single nucleotide polymorphisms.

Figure 3 presents a scatter plot correlating lymphocyte subgroups and sepsis, and the subsequent pleiotropy test yields no evidence of horizontal pleiotropy. Figure 4 illustrates the result of a “leave-one-out” sensitivity analysis. The findings indicate that, upon sequential exclusion of each SNP, the IVW analysis outcomes from the remaining SNPs align closely with those derived from an inclusive analysis of all SNPs. Figure 5 is a funnel plot demonstrating that the dispersion of the causal association effect is largely symmetrical, which suggests there is no significant bias in the results.

## 4. Discussion

This study established a causal correlation between the absolute count of CD4 Treg within lymphocyte subgroups and sepsis risk, aligning with prior research findings. Earlier studies demonstrated a close connection between the levels of lymphocyte subgroups in sepsis patients’ peripheral blood and their prognosis.^[31]^ Despite abundant research on sepsis pathogenesis, its etiology remains uncertain. Emphasizing the role of immunity on the risk, occurrence, progression, and sepsis fatality, a substantial amount of recent research is notable. The apoptosis or decrease of CD4+ T cells indeed assumes a crucial role in sepsis progression. The induction, activation and level improvement of CD4+ T cells have a significant implication on the prognosis of sepsis.^[32,33]^ Dendritic cells or monocytes stimulate the activation of CD4+ T cells, which in turn release immunomodulatory cytokines and orchestrate the function of cytotoxic CD8+ T cells.^[8]^ Through flow cytometry, Zheng et al^[34]^ assessed T lymphocyte subgroups and the influence of vitamin D on children suffering from sepsis. Their findings revealed that the CD4+ T lymphocyte subgroup differentiation rate was substantially lower in sepsis patients administered with oral vitamin D compared to a healthy control group (*P* < .01). This suggests that vitamin D potentially enhances the prognosis of sepsis patients by modulating T lymphocyte subgroups and inflammatory factors. A prospective study inspecting the effect of lymphocyte abnormalities on sepsis or critically ill patients observed a reduction in lymphocytes, T cells and NK cells in swollen and intensive care unit (ICU) patients unrelated to sepsis. However, lymphocyte depletion and T cell consumption associated with ICU admission correlated with increased mortality rates. Interestingly, the lymphocyte and T cell count could rapidly recover within 48 hours in surviving critically ill patients,^[35]^ consistent with findings from large multicenter studies.^[36]^ The downregulation in CD4+ and CD8+ immunity may underscore the sepsis occurrence and mortality.^[37]^ Given this, 120 sepsis patients were segregated into 3 groups: sepsis, severe sepsis and septic shock, with 40 individuals in each group, for a clinical observation study. The correlation of T lymphocyte subgroup, IL-6, and PCT with sepsis severity was examined. As the severity of sepsis escalated, levels of CD3+, CD4+, and CD4+/CD8+ correspondingly diminished in the 3 patient groups, while IL-6 and PCT levels exhibited a positive correlation with sepsis severity. Higher levels of CD4+ and the ratio of CD4+/CD8+ posttreatment correlated with improved patient recovery.^[38]^ Accordingly, B cells support humoral immune responses.^[39]^ In Wilson et al’s^[40]^ observational study, programmed death protein 1 levels in B cells and CD4+ T cell memory subgroups in sepsis patients were notably higher than in healthy individuals. NK cell subgroups contribute to cytotoxic activity and cytokine release. Li et al^[10]^ implemented a retro- and prospective cohort study to authenticate the impact of immune imbalance on sepsis-related delirium. In the retrospective component, it was found that the lymphocyte count in delirium patients at ICU admission was markedly higher than the non-delirium group. However, no significant correlations were observed in terms of B lymphocyte, CD4 cell and CD8+ T lymphocyte counts between SAD and non-SAD patients in the prospective study. These findings suggest that NK cell count may serve as an important predictor in the elderly patient cohort of SAD.^[41]^ Thus, previous studies converge on the link between lymphocyte subgroups and the onset of sepsis.

The present study boasts several merits. Primarily, it pioneers the investigation of the causal relationship between serum lymphocyte subgroups and the risk of sepsis from a genetic variation perspective using 2-sample MR. Second, in comparison to randomized control trials, MR research design is less prone to confounding factors and reverse causality. Lastly, the data utilized in this study stringently abides by the 3 central assumptions of MR, thereby evading weak instrument bias.

Nevertheless, the study has its limitations. First, despite the fact that the participants of the GWAS were derived from the Sardinian population, discrepancies due to population stratification might still be present. Consequently, these findings may not be fully applicable to individuals of non-European descent. Second, while scrutinizing SNPs related to lymphocyte subgroups, the data significance threshold was relaxed to 5 × 10^−6^. Although a nominal *P* value of < 5 × 10^−8^ is considered genomically significant, fewer SNPs were detected at this threshold, thus necessitating a reasonable reduction of the threshold. This could potentially influence the findings to a certain extent. Third, while the study validated the association between CD4 cell count levels in the lymphocyte subgroups and the risk of sepsis from a genetic perspective, the specific mechanism underlying this is complex. Future research is required for further validation.

In conclusion, the study ascertains the causal correlation between CD4 cell count levels and sepsis risk from a genetic standpoint. These findings may offer new therapeutic targets for diagnosing and treating sepsis.

## Acknowledgments

The authors extend their appreciation to the IEU Open GWAS database (https://gwas.mrcieu.ac.uk/) for its invaluable contributions to our research.

## Author contributions

**Data curation:** Jing Chen.

**Formal analysis:** Jing Chen.

**Writing**—**original draft:** Jing Chen, Rong Hui Wang, Fu Kui Zheng, Zu Lu Liu.

**Writing**—**review & editing:** Sheng Xie.

**Validation:** Jun Jun Xiang.

**Methodology:** Qiao Ming Huang, Qiu Lan Mo.

**Supervision:** Qiu Gui Wei.

## Supplementary Material


