# Supplementary material for: Causal relationship between lymphocyte subsets and the risk of sepsis: A Mendelian randomization study
Source: Medicine (Baltimore). 2024 Oct 4;103(40):e39871. doi: 10.1097/MD.0000000000039871 (PMC11460878; doi:10.1097/MD.0000000000039871)
Supplement: Supplementary file 1 [file medi-103-e39871-s001.docx]

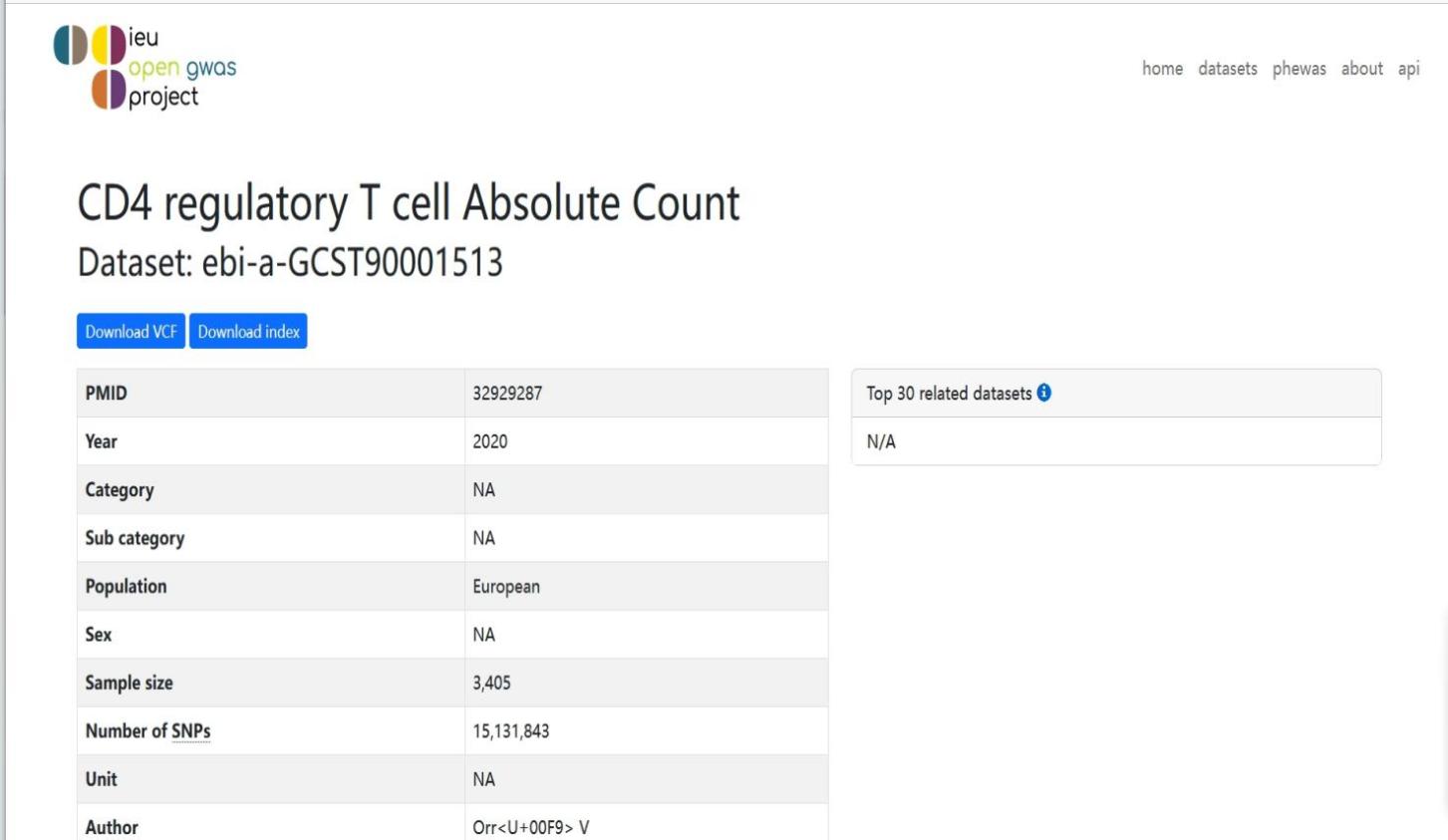


Fig1-a.CD4 regulatory T cell Absolute Count


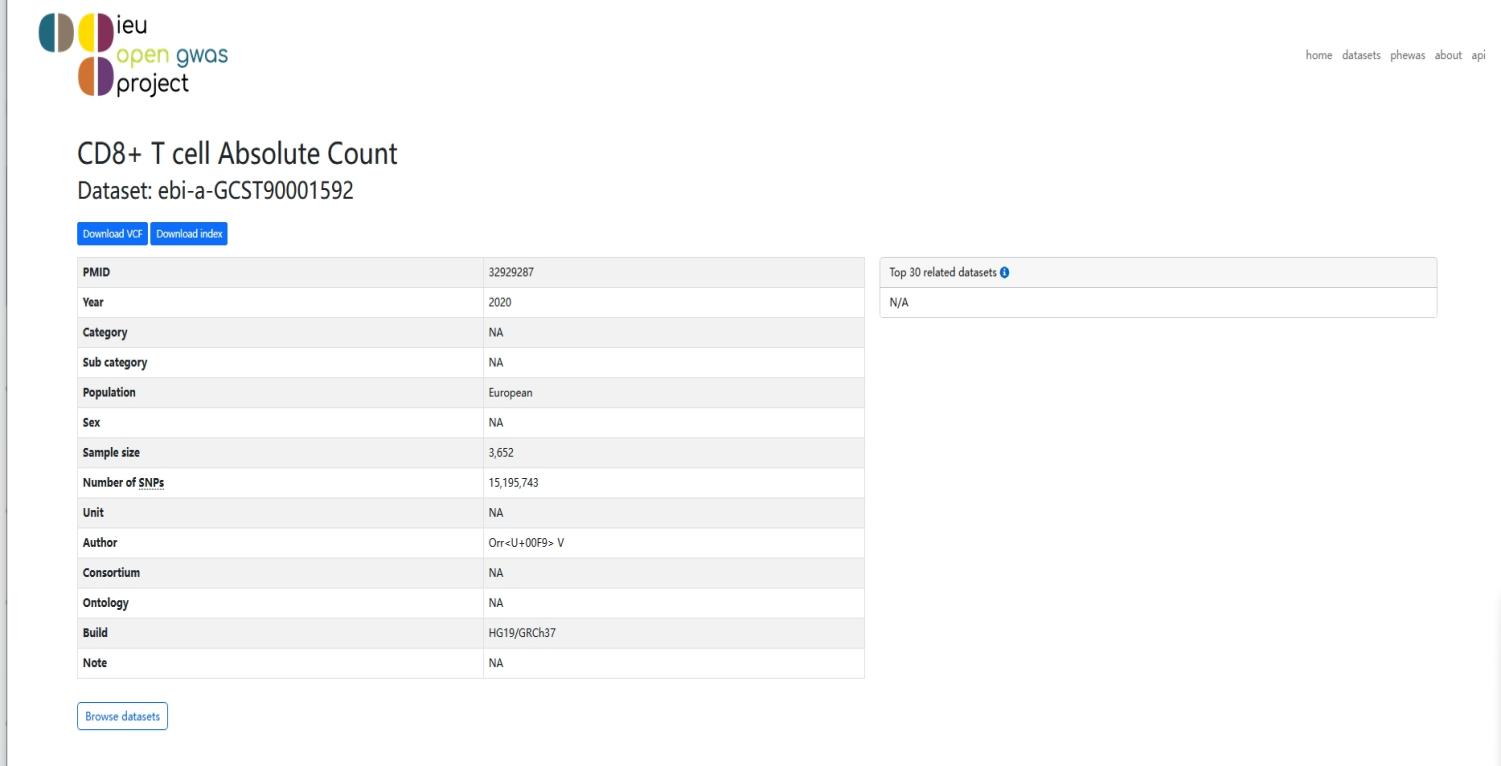


Fig1-b.CD8+ T cell Absolute Count


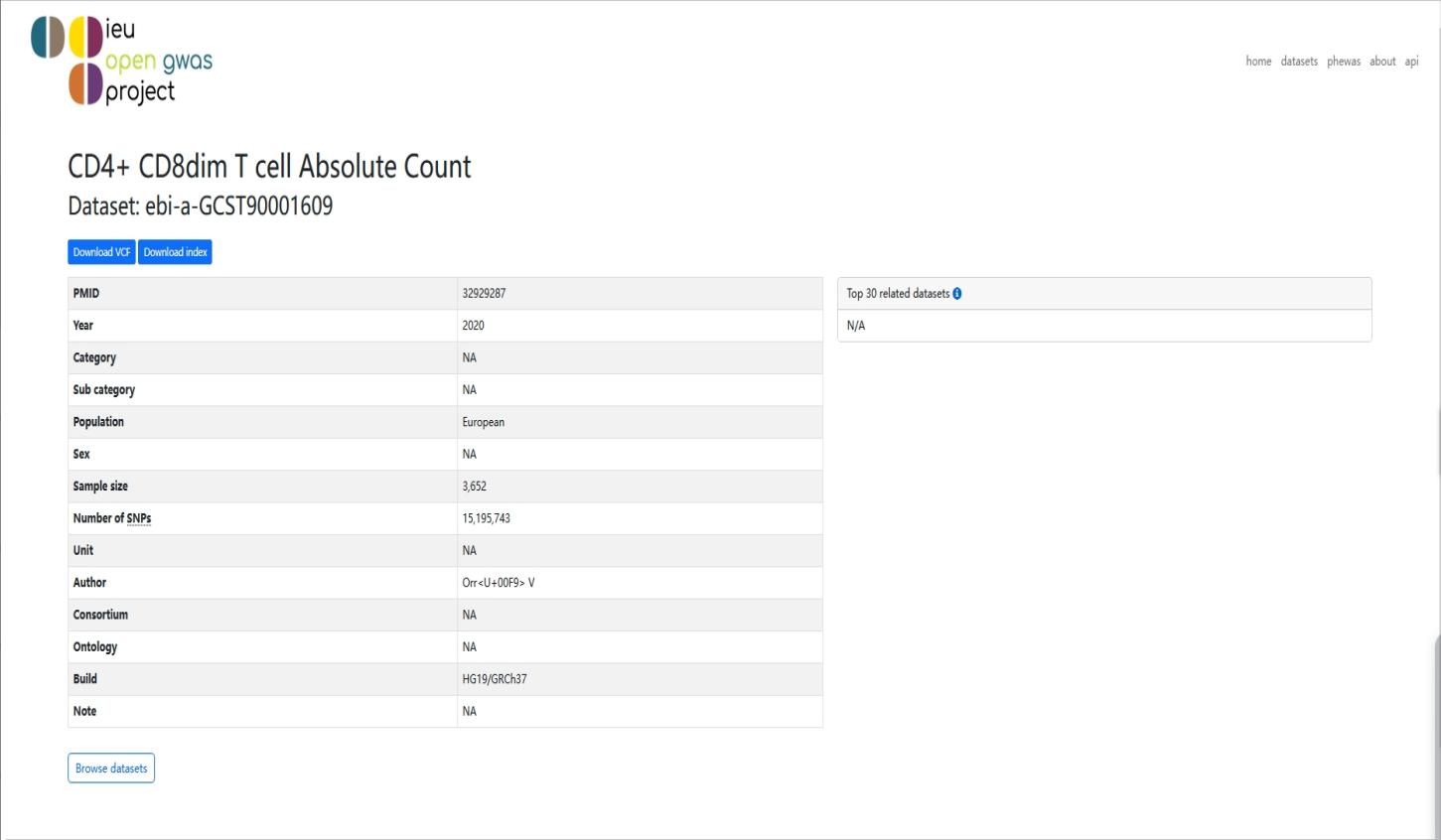


Fig1-c.CD4+ CD8dim T cell Absolute Count


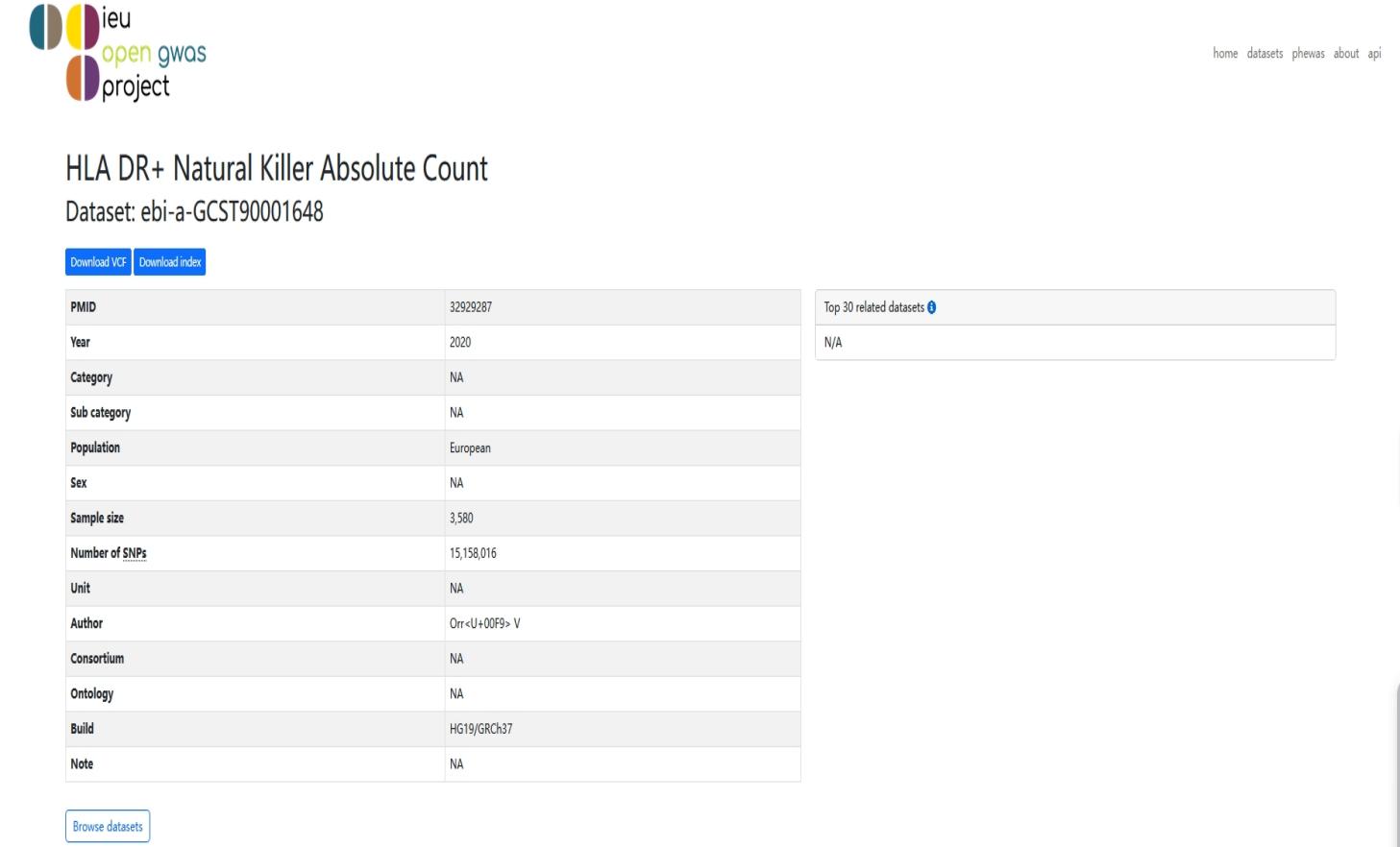


Fig1-d.HLA DR+ Natural Killer Absolute Count


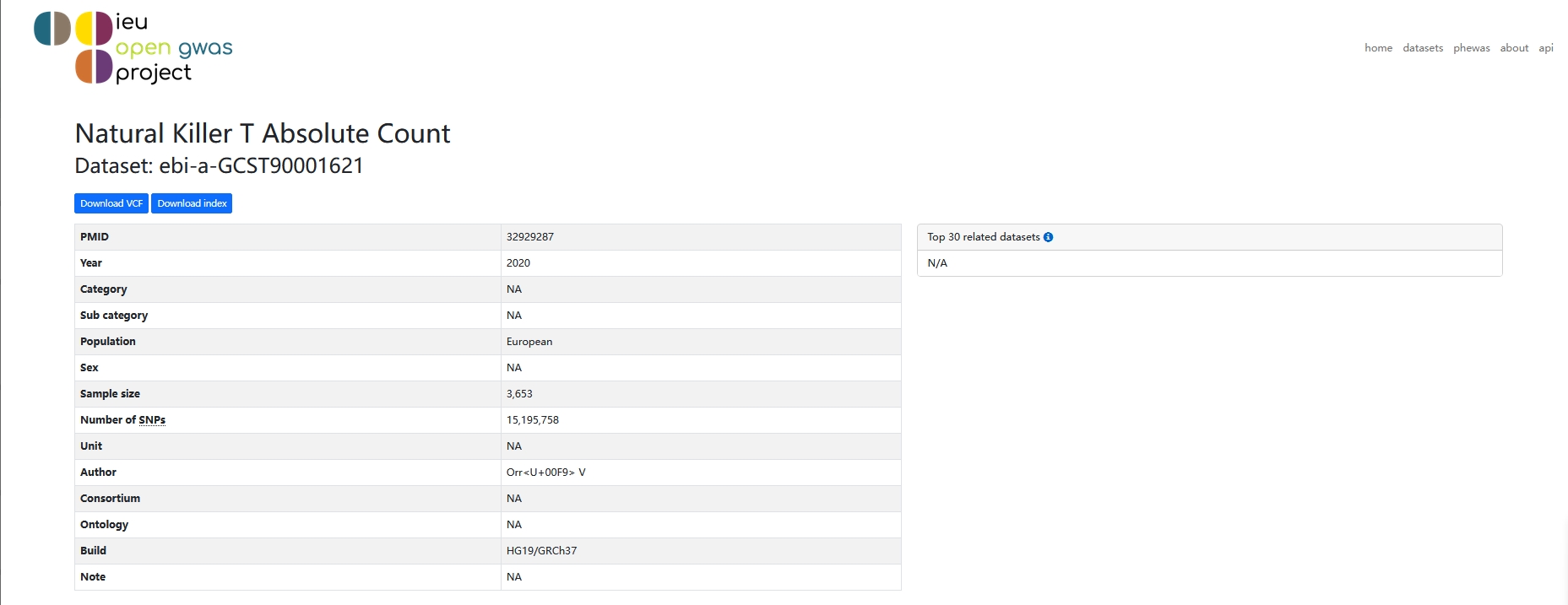


Fig1-e.Natural Killer T Absolute Count


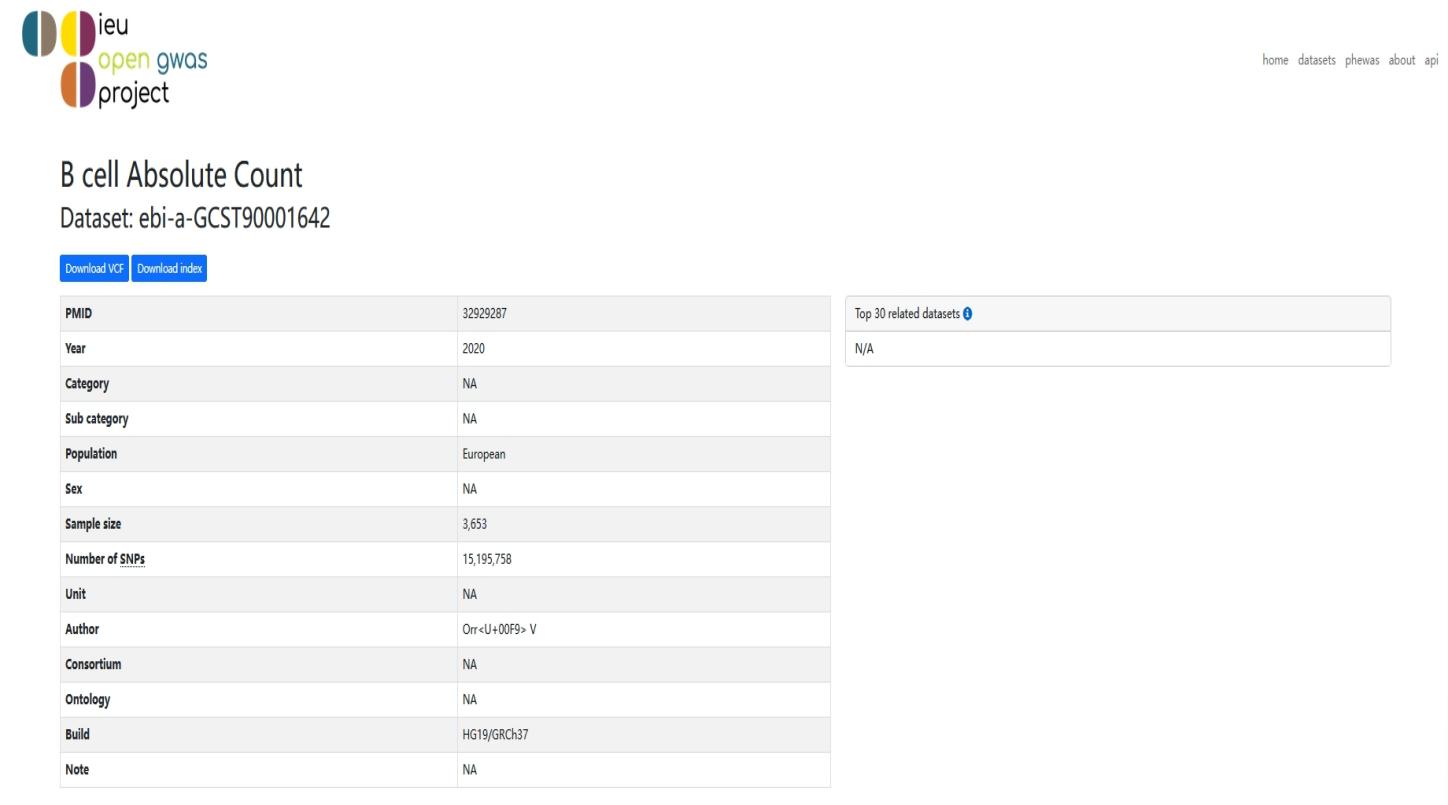


Fig1-f.B cell Absolute Count

Supplemental Digital Content for Figure1.Figures that show specific information on six exposure factors.Fig1-a, Fig1-b, Fig1-c, Fig1-d,Fig1-e,and Fig1-f,Which respectively show the CD4 regulatory T cell Absolute Count, CD8+T cell Absolute Count, CD4+CD8dim T cell Absolute Count, HLA DR+Natural Killer Absolute Count, Natural Killer T Absolute Count, and B cell Absolute Count among the six exposure factors. The above charts all contain information such as Sample size, Number of SNPS, Consortium Population, Sex, Year, etc. These information are sourced from https://gwas.mrcieu.ac.uk.
